# Water near its Supercritical Point and at Alkaline pH for the Production of Ferric Oxides and Silicates in Anoxic Conditions. A New Hypothesis for the Synthesis of Minerals Observed in Banded Iron Formations and for the Related Geobiotropic Chemistry inside Fluid Inclusions

**DOI:** 10.1007/s11084-018-9560-y

**Published:** 2018-08-08

**Authors:** Marie-Paule Bassez

**Affiliations:** Institut de Technologie, Université de Strasbourg, 67400 Illkirch-Graffenstaden, Strasbourg, France

**Keywords:** Anoxic iron oxidation, Abiogenic ferric iron, High-subcritical water, High pH, Ferrous/ferric iron, Ferric oxide hydroxides, Ferric oxides, Ferric silicates, Amorphous silica, Banded Iron Formations, Hamersley Group, Transvaal Supergroup, Prebiotic matter, Fluid inclusions, Amino-acids, Geobiotropy, Geobiotropic chemistry, Geochemical origin of life, Theoretical & experimental work, Raman analysis

## Abstract

An alternative hypothesis for the origin of the banded iron formations and the synthesis of prebiotic molecules is presented here. I show the importance of considering water near its supercritical point and at alkaline pH. It is based on the chemical equation for the anoxic oxidation of ferrous iron into ferric iron at high-subcritical conditions of water and high pH, that I extract from E-pH diagrams drawn for corrosion purposes (Geophysical Research Abstracts Vol 15, EGU2013–22 Bassez 2013, Orig Life Evol Biosph 45(1):5-13, Bassez 2015, Procedia Earth Planet Sci 17, 492-495, Bassez 2017a, Orig Life Evol Biosph 47:453-480, Bassez 2017b). The sudden change in solubility of silica, SiO_2_, at the critical point of water is also considered. It is shown that under these temperatures and pressures, ferric oxides and ferric silicates can form in anoxic terrains. No Fe^II^ oxidation by UV light, neither by oxygen is needed to explain the minerals of the Banded Iron Formations. The intervention of any kind of microorganisms, either sulfate-reducing, or Fe^II^-oxidizing or O_2_-producing, is not required. The chemical equation for the anoxic oxidation of ferrous iron is applied to the hydrolyses of fayalite, Fe_2_SiO_4_ and ferrosilite, FeSiO_3_. It is shown that the BIF minerals of the Hamersley Group, Western Australia, and the Transvaal Supergroup, South Africa, are those of fayalite and ferrosilite hydrolyses and carbonations. The dissolution of crustal fayalite and ferrosilite during water-rock interaction needs to occur at T&P just below the critical point of water and in a rising water which is undersaturated in SiO_2_. Minerals of BIFs which can then be ejected at the surface from venting arcs are ferric oxide hydroxides, hematite, Fe^III^-greenalite, siderite. The greenalite dehydrated product minnesotaite forms when rising water becomes supersaturated in SiO_2_, as also riebeckite and stilpnomelane. Long lengths of siderite without ferric oxides neither ferric silicates can occur since the exothermic siderite formation is not so much dependent in T&P. It is also shown that the H_2_ which is released during hydrolysis/oxidation of fayalite/ferrosilite can lead to components of life, such as macromolecules of amino acids which are synthesized from mixtures of (CO, N_2_, H_2_O) in Sabatier-Senderens/Fischer-Tropsch & Haber-Bosch reactions or microwave or gamma-ray excitation reactions. I propose that such geobiotropic synthesis may occur inside fluid inclusions of BIFs, in the silica chert, hematite, Fe^III^-greenalite or siderite. Therefore, the combination of high-subcritical conditions of water, high solubility of SiO_2_ at these T&P values, formation of CO also at these T&P, high pH and anoxic water, leads to the formation of ferric minerals and prebiotic molecules in the process of geobiotropy.

## Introduction

Below I present an alternative to the usually accepted theory that ferric oxides form in the presence of UV light, oxygen, or microorganisms, introducing my 2013 proposition that they form through anoxic oxidation of Fe^II^ in high-subcritical water and high pH, and that this process can lead to the formation of prebiotic molecules in the process of geobiotropy. I apply this anoxic oxidation to banded iron formations.

Ferrous and ferric oxides have been extensively studied. Books discuss their structures, properties, occurrences, formations and transformations in geology and biology, including the action of iron(II)-oxidizing and iron(III)-reducing bacteria, IOB and IRB (Faivre 2016, Cornell and Schwertmann 2006, and refs herein). Articles discuss the close association of iron with biological systems, for instance in Taylor and Konhauser 2011, or Posth et al. 2018, and refs hereins). Since 2013, I show in conferences and articles that ferric compounds can form under anoxic conditions, at high T ~300°C–350 °C, high P ~10MPa–25 MPa and high pH ~9.5–14. This proposition is based on a new redox equation that I extract from E-pH diagrams drawn for the system Fe-H_2_O (Cook and Olive 2012). The diagrams are posted at the URL address in Bassez (Bassez 1998–2018: La Géobiotropie). This equation which represents the oxidation of Fe^II^ into Fe^III^ in the absence of oxygen and with the release of H_2_, led to the thermodynamic studies of hydrolyses and carbonations of silicates (Bassez 2013–Bassez 2017a, b) and to the new concept of geobiotropy (Bassez 2016a, b, 2017a, b). The understanding that synthesis of ferric compounds from ferrous compounds can occur in anoxic conditions, opens wide areae of new interpretations of chemical processes on Earth and in the Universe. In this article I show one part of this new domain: how the alkaline anoxic abiotic oxidation of ferrous iron can bring new insights into the understanding of the Banded Iron Formations and the origin of life.

The products of the hydrolyses and carbonations of the iron-rich olivine and pyroxene silicates, fayalite and ferrosilite, are identified considering water in its high-subcritical state and silica, SiO_2_, solubility in its discontinuous behavior at the critical point of water. Further on, these products can dehydrate into particular amphiboles and phyllosilicates when solutions are supersaturated in SiO_2_ at high-subcritical conditions of water and these amphiboles and phyllosilicates are those found in the layers of BIFs.

I observe that during the interaction between water and iron containing rocks at high T high P high pH, the rock produces electrons which are captured by water, releasing thus ferric iron in the absence of oxygen, and releasing also H_2_ which can be used in prebiotic reactions. This process of evolution of rocks and their mineral contents, which occurs in symbiosis with the synthesis of prebiotic molecules, is conceptualized within the word geobiotropy. It is a concept which differs from the concept of catalysis where minerals are recovered unchanged after the reactions. I first proposed this action of rocks as reactants and not only as catalysts in Bassez (2003): *“Therefore, specific reactions might occur in particular cavities, while cavity walls might act as catalysts or reactants.”* This idea started to be developped in Bassez (2008, 2009a, b), considering a chemical evolution with a geological origin. Experiments were proposed with the rock peridotite as reactant. In the contrary to geobiology which is the study of how microbial processes leave imprints on rocks, geobiotropy is the study of the transformation of rocks during their interaction with water and carbonated water while leading towards the formation of prebiotic molecules. In the search for life, geobiology studies the signatures that life leaves on rocks and geobiotropy the signatures that rocks leave on themselves during their evolution towards prebiotic molecules. Prebiotic chemistry instead of life may have left its signature in the Archean era, before the Great Oxidation Event, as it is shown here for BIFs. Archean prebiotic chemistry may proceed inside the closed systems that are fluid inclusions possibly enclosed in the silica, hematite, Fe^III^-greenalite and siderite of BIFs and/or hydrothermal terrains.

Thus, neither UV light, nor oxygen, nor sulfate-reducing bacteria, nor iron-oxidizing bacteria, nor O_2_-producing cyanobacteria, are necessary to explain the constituents of BIFs. Instead minerals of BIFs and/or hydrothermal rocks can form in alkaline anoxic high-subcritical water and may be at the origin of the formation of prebiotic molecules and life. The continuity from rocks to the components of life seems best illustrated by the painting of Newton that William Blake produced in the years 1795–1805 and that I interpret as the evolution from rocks to life and intelligence.

## Calculations and Methods of Theoretical and Experimental Analysis

In order to demonstrate scientifically the idea of continuity from rock to life, I start with the analysis of E-pH Pourbaix diagrams drawn for corrosion purposes in 2012, by William G. Cook and Robert P. Olive, for the system Fe-H_2_O at high-subcritical, low-supercritical conditions, 300 °C–350 °C, high P 10 MPa–25 MPa.

A private communication with W. Cook in September 2016 confirmed the drawings. I observed that at high pH, the redox line located between the Fe(OH)_4_^-^ and Fe(OH)_3_^-^ ions is positioned below the redox line H^+^/H_2_. This means that the redox potential line of the H^+^/H_2_ couple is higher than the redox potential line of the Fe^3+^/Fe^2+^ couple and that the H^+^/H_2_ couple oxidizes the Fe^3+^/Fe^2+^ couple. In other words, when the Fe^2+^ ion or the divalent form of iron, Fe^II^, is in contact with alkaline near supercritical water, water transforms into H_2_ and ferrous iron transforms into ferric iron in the absence of oxygen. This is represented by the Eq. (1) of Table 1. This redox equation is then applied to chemical equations of dissolution of rocks which contain olivine and pyroxene. Calculations of the thermodynamic functions enthalpies and free enthalpies for the endothermic and exothermic hydrolyses and carbonations of Fe^II^,Mg-silicates and Fe^II^-monosulfides, together with the analysis of thermodynamic E-pH diagrams were reported earlier (Bassez 2013) and published in articles (Bassez 2015–2017a, b).

The new Eq. (1) of anoxic oxidation of ferrous iron is applied here to the minerals observed in BIFs. The method consists in the observation that silica, SiO_2_, which is produced in the hydrolysis and carbonation of fayalite and ferrosilite, has the property of being highly soluble under the same conditions of T and P that lead to the anoxic oxidation of ferrous iron into ferric iron. The state of water considered is high-subcritical, that I write hsc. I observe a discontinuous behavior in the solubility of silica, SiO_2_, at the critical point of water. The solubility falls to almost zero just above the critical point and is high just below the critical point. I apply this observation to the anoxic hydrolysis and carbonation of the Fe^II^-rich endmembers of olivine, fayalite Fe^II^_2_SiO_4_ and pyroxene, ferrosilite Fe^II^SiO_3_, and to the dehydration products of greenalite, considering that the interacting water may already contain silica. The validity of the equations showing the minerals which form when water is in the high-subcritical state is discussed through several recent experiments conducted by other authors. These experiments were not reported in the earlier articles.

The proposed prebiotic chemistry inside fluid inclusions is also based on this anoxic oxidation of ferrous iron. The Fe^II^-olivine and -pyroxene containing rocks evolve in alkaline hsc water to form Fe^III^-oxides and -silicates together with H_2_ which, from a product of inorganic water-rock interaction, becomes a reactant in organic reactions.

The experimental analysis of the content in minerals and organic matter of Archean rocks is conducted with Raman spectroscopy and imaging. The instrument used is the Witec alpha300 confocal microscope operated with a green 532 nm laser light and located in the department of geology in the university of Johannesburg, South-Africa.

## Theoretical Results with Application to BIFs and Geobiotropy

### High-Subcritical Anoxic Alkaline Water for the Formation of Ferric Iron and Ferric Minerals. Analyses of E-pH and Solubility Diagrams and of Published Experimental Results

Table 1 shows the chemical equations for the anoxic formation of ferric iron (1) and the consecutive minerals that can be classified as ferric oxides/hydroxides, silica, carbonates and ferric silicates. Details of these formations are described below in connection with the minerals of the Banded Iron Formations, BIFs, which are described in detail in the next chapter.

**Table 1:**
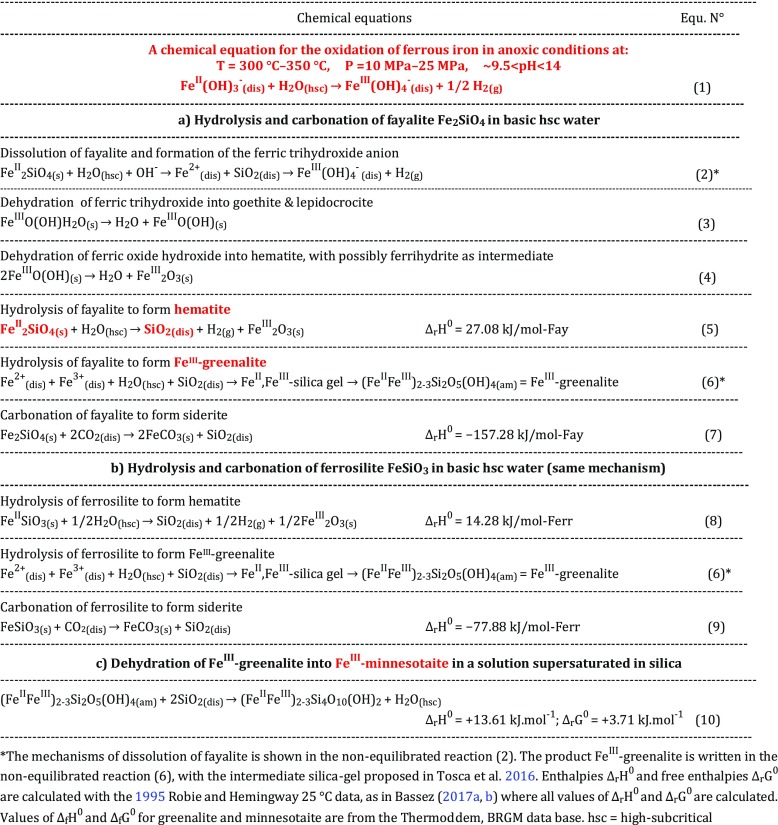
Hydrolysis/Oxidation of ferrous iron into ferric iron in anoxic alkaline high-subcritical water. Mechanism of dissolution of fayalite and ferrosilite to form hematite

#### Formation of Ferric Iron in Anoxic High-Subcritical Water

The iron hydroxides species Fe(OH)_3_^-^ and Fe(OH)_4_^-^ appear in the Pourbaix diagrams, published by Cook and Olive in 2012, at high-alkaline pH ~9.5–14, high T 300 °C–350 °C and high P 10 MPa–25 MPa. From these diagrams, I write the chemical Eq. (1) (Bassez 2013–Bassez 2017a, b), which represents the anoxic oxidation of ferrous iron at high-subcritical (hsc) conditions of water (supercritical conditions of pure water are: Tc = 374 °C, Pc = 22.1 MPa). Fe^II^ is oxidized into Fe^III^ by loss of one electron and H_2_O is reduced to 1/2 H_2_ by gain of one electron. While Cairns-Smith proposed that in Precambrian times, *“that was the great period of ferric sedimentation in the form of …BIFs”,* UV light can oxidize Fe^II^ in acidic water (Cairns-Smith 1978), I propose that it is alkaline water near its critical point which oxidizes Fe^II^ into Fe^III^ and plays the role of electron-acceptor.

#### Dissolution of Fayalite and Formation of Dissolved Fe^2+^

As a consequence of Eq. (1), the ferrous iron of fayalite and ferrosilite should oxidize in anoxic alkaline subcritical water to form the dissolved ferric species Fe(OH)_3_OH^-^. However, Fig. 4 in Cook and Olive (2012) shows that the pH of water is acidic at high-subcritical T&P (5.8 at 350 °C & 25 MPa, 5.6 at 350 °C & 50 MPa) and that it increases highly in the supercritical state (9.8 at 540 °C & 25 MPa, 7.4 at 540 °C & 50 MPa). Thus, pH-controlling reagents need to be present to make hsc water alkaline, for instance ammonium hydroxide which produces pH 10.6 at 25 °C in a 0.01 N solution (PubChem 2018) and which could be locally outgassing from the Earth’s interior in Archean times. However, at the T&P of hsc water, ammonia is in the supercritical, sc, state (Tc = 132 °C, 11.33 MPa) with a structure where the intermolecular hydrogen bonds of the liquid *“are strongly attenuated”* (Tassaing et al. 2010). Therefore pH values should be different than those in liquid ammonium hydroxide aqueous solutions at 25 °C. Nonetheless, sc ammonia can most probably associate with Fe^2+^ to form complexes which may drive the dissolution of fayalite. Indeed, the kinetics of fayalite dissolution in water was studied at acid pH 1.11–2.70, 30 MPa and 50 °C to 100 °C by Daval et al. (2010) who propose that “*as for many other minerals, organic ligands are able to promote the dissolution of fayalite via surface complexation*” and that Fe^2+^ can associate with two acetate ligands to form Fe(CH_3_COO)_2_.4H_2_O. *“This solvation…would explain the observed enhancement of the dissolution rate”.* In alkaline solutions, the Fe(II)-ammonia complex Fe(NH_3_)_a_^2+^ (a = 1,2) is known to exist and also the hydroxoamminocomplex Fe(OH)_n_(NH_3_)_a_^(2-n)+^ (*n* = 1,2,3) (Ziemniak et al. 1995 and ref. herein). Thus, ammonia in hsc water should lead to alkaline pH and Fe^II^-ammonia complexes which help the dissolution of fayalite.

KOH could also set alkaline pH. For example, Fig. 24 in Macdonald et al. (1992) shows that a solution of 0.01 M KOH produces pH ~9 at 275 °C and ~9.5 at 374 °C. However, the best candidate for basic and ultrabasic water is NaOH as observed in samples from springs issuing from ultramafic rocks in California and Oregon (Barnes et al. 1967) where *“waters of two chemically distinctive types are found.”* The huge difference between the moderately alkaline water (pH 8.3–8.6) and the ultrabasic water (pH 11.2–11.8) is the abundance of Na^+^ which is up to ~60 times higher in the ultrabasic water. NaOH could arise from the dissolution of the mineral disodium carbonate named natrite Na_2_CO_3_ (Khomyakov 1983; Arakcheeva et al. 2010) which forms strongly alkaline aqueous solutions. At 25 °C, a solution of Na_2_CO_3_, 1 wt%, has a pH of 11.37 (PubChem 2018). Other carbonates can be envisioned: thermonatrite Na_2_CO_3_.H_2_O, natron Na_2_CO_3_.10H_2_O and also dipotassium carbonate K_2_CO_3_. I am currently studying modern analogs of alkaline waters which are found in basic or soda lakes. For instance, the lake Magadi in Kenya shows pH 9 and large deposits of trona NaCO_3_.NaHCO_3_.2H_2_O and sodium-aluminium silicate gels (Eugster and Jones 1968).

The dissolution of fayalite in hsc water should also be induced considering the solubilities of amorphous and crystalline silica which both increase with T and P apart from an exception at the critical point of water as shown below in the paragraph on silica. In hsc water, both amorphous and crystalline silica dissolve in equal amounts. Thus, fayalite should dissolve easily in hscw conditions. In extra, the more fayalite dissolves, the more goethite forms and the more silica is adsorbed on goethite (Siever and Woodford 1973; Sigg and Stumm 1981), inducing more dissolution of fayalite. The same process of adsorption is observed when a silica-rich layer covers a hematite nanocrystal core, as shown in Fig. 6 of the experiment conducted by Qafoku et al. (2012) which is described below in the section on *Related Laboratory Experiments* of the present chapter. The effect of hydrolysis of fayalite to form goethite, hematite and silica is combined to the adsorption of silica on goethite and hematite which decreases the concentration of silica in solution, and consequently further promotes fayalite dissolution. Fayalite should dissolve until silica saturation is achieved, as confirmed by the experiment conducted by Karasek et al. (2013a) on fused silica capillaries, described below in the section on *Dissolution of Silica* of the present chapter*.*

Therefore, some local environmental conditions of T, P, pH and composition of hsc water may combine in order that fayalite & ferrosilite dissolve in alkaline high-subcritical water. The observation of the dissolution of fayalite in supercritical CO_2_ (Qafoku et al. 2012) is described below in the section on laboratory experiments.

#### Formation of Iron Oxides Hydroxides

Following the dissolution of fayalite, the Fe(OH)_3_OH^-^ species can form. Fe(OH)_3_OH^-^ is the anion form of ferric trihydroxide Fe(OH)_3_, which can be written as FeO(OH)H_2_O, which can dehydrate into the ferric oxide hydroxides α- and γ- FeO(OH) named respectively goethite and lepidocrocite (Eq. 3), and into the ferric oxide hematite α-Fe_2_O_3_ (Eq. 4). Magnetite Fe_3_O_4_ is a secondary product which forms at ~400°C and above as described in Bassez 2017b on the basis of published experiments. Other experiments show that magnetite can be produced from siderite such as in siderite decomposition at T ~450°C 50 MPa (French and Rosenberg 1965) or siderite hydrolysis with release of H_2_ at 300 °C, 50 MPa (Milesi et al. 2015).

The plausible dehydration of iron hydroxides into iron oxides is reinforced by the observations made by Adschiri and co-workers between 1992 and 2001, that *“fine metal oxide particles form when metal nitrates are contacted with supercritical water in a flow system.”* The authors *“postulated”* that *“the fine particles were produced because supercritical water causes the metal hydroxides to rapidly dehydrate before significant growth takes place... Processing in Scw increases the rate of dehydration.”* (Byrappa and Adschiri 2007 p.136 and refs herein). In these experiments, hematite particles formed at 400 °C, 35 MPa with high dehydration rates. They were spherical and ~ 20 to ~ 50 nm in size. *“We think the high reaction rates obtained in this experiment are due to the elevated reaction temperature, the high diffusivity of the reactants in supercritical water, and the fast dehydration reaction of the fine hydrous oxide particles.”* (Adschiri et al. 1992). In these experiments, the dehydration of iron oxide hydroxides into oxides occurs in supercritical water. However, the properties of high-subcritical water approaching those of low supercritical water (Cook and Olive 2012) it is most probable that oxides form also from oxide hydroxides at subcritical conditions.

The intermediate poorly ordered ferrihydrite Fe_5_HO_8_.4H_2_O can also form prior to final hematite, α-Fe_2_O_3_ as experimentally demonstrated in Rzepa et al. (2016) where the transformation of ferrihydrite to hematite is observed at 300–350 °C.

Therefore, high-subcritical water allows the formation of ferric iron in anoxic conditions and also increases the rates of formation of hematite from goethite and lepidocrocite, with an intermediate compound which can be ferrihydrite. As it is described in the chapter on Banded Iron Formations, hematite can be a primary product, in this proposed process of formation.

#### Formation of Fe^III^-Silicates

The silicate greenalite Fe^II^_3_Si_2_O_5_(OH)_4_ is the iron equivalent of the serpentine chrysotile, Mg_3_Si_2_O_5_(OH)_4_, which forms by the action of water on the Mg-endmembers of olivine and pyroxene with production of brucite Mg(OH)_2_ (Bassez 2017a&b). However, Eq. (6) is written here, not by analogy with the formation of chrysotile, but considering the structures which are experimentally observed when dissolved Fe^2+^ is mixed with silica SiO_2_ in anoxic aqueous solutions at 25 °C. The formation of a Fe(II)-silicate gel is observed. Tosca et al. (2016) describe a *“hydrated, disordered Fe-silicate “gel“ phase…Subsequent dehydration and structural rearrangement within the gel phase lead to the formation of nanoparticle domains that exhibit short-range order…producing the structure of greenalite”.* In 1998, Guggenheim and Eggleton observed with Transmission Electron Microscopy that greenalite is a modulated 1:1 phyllosilicate: *“the octahedrally coordinated Fe…form trioctahedral sheets. Six-member rings of tetrahedra link to form triangular islands…The tetrahedra show limited short-range order…but long-range disorder. Linkages of tetrahedra between islands are apparently complety disordered.”* Diagrams for the activity of Fe^2+^ as a function of pH, drawn at 25 °C (Fig.12 in Tosca et al. 2016, show that greenalite starts to nucleate at pH 9 in a much lower content of dissolved Fe^2+^ than at pH 7*.* Thus, greenalite can nucleate in alkaline solutions containing a small amount of Fe^2+^.

As described in Bassez 2017a & b, chrysotile can form as (Mg,Fe^II^)_3_Si_2_O_5_(OH)_4_ or (Al,Fe^III^)_2_Si_2_O_5_(OH)_4_, thus incorporating the ferric iron which is produced in anoxic alkaline hsc water. In the same process, greenalite can incorporate Fe^III^ to form (Fe^II^,Fe^III^)_2-3_Si_2_O_5_(OH)_4_ and cronstedtite can also form as Fe^II^_2_Fe^III^(SiFe^III^)O_5_(OH)_4_. Chrysotile, greenalite and cronstedtite belong all three to the serpentine group. Fe^III^-greenalite synthesized in alkaline hsc water can thus be a primary product in BIFs. This chemical process of greenalite formation finds confirmation in the very recent microscopic and spectroscopic observations of nanoparticle-bearing samples from ~2.5 Ga BIFs and ferruginous cherts hosted in well-preserved cores from Western Australia and South Africa (Johnson et al. 2018). They conclude that greenalite contains 10–20% Fe(III) and should be considered as a primary mineral of the BIFs.

Other silicates can also form as primary minerals of BIFs. Indeed, chrysotile dehydrates into talc Mg_3_Si_4_O_10_(OH)_2_ when the solution is supersaturated in SiO_2_. As chrysotile, greenalite can dehydrate, when in a solution supersaturated in SiO_2_, into minnesotaite Fe^II^_3_Si_4_O_10_(OH)_2_ or (Fe^II^,Fe^III^)_2-3_Si_4_O_10_(OH)_2_ which is the iron-rich talc, following Eq. (10). Dehydration of greenalite is calculated here to be slightly endothermic. It can proceed easily in subcritical water. Thus, minnesotaite can form as a primary product in hsc water supersaturated in silica. Since both reactions of hydrolysis and carbonation of fayalite produce silica and that the solubility of silica is high in hsc water, hsc water can be supersaturated in silica, inducing easily the synthesis of minnesotaite.

Further dehydration of greenalite can most probably lead to riebeckite and stilpnomelane. Indeed, the sodium iron silicates hydroxides of the amphibole group, riebeckite Na_2_(Fe^II^_3_,Fe^III^_2_)Si_8_O_22_(OH)_2_, grünerite Fe^II^_7_Si_8_O_22_(OH)_2_, and the potassium iron magnesium silicate oxide hydroxide stilpnomelane which forms a series of phyllosilicates with the formula K(Fe^II^,Mg,Fe^III^)_8_(Si,Al)_12_(O,OH)_27_.n(H_2_O) as reported in Haugaard et al. 2016, comprise much less hydroxide (-OH) groups than minnesotaite. To my knowledge, they can thus be considered as highly dehydrated products of greenalite which form most probably in aqueous solutions supersaturated in silica. The experiment of Tosca et al. (2016) mixing Fe^2+^ and SiO_2_ was conducted at 25 °C. It seems most interesting to conduct the same kind of experiment in alkaline high-subcritical water with high level of silica, and observe if the hydrated disordered Fe-silicate gel structurally transforms into stilpnomelane when alkalinity is controlled by KOH and into riebeckite when alkalinity is controlled by NaOH. As greenalite and minnesotaite, stilpnomelane and riebeckite could also incorporate Fe^III^ in their structures, since they would form first in alkaline hsc water (conditions for formation of Fe^III^) and second from dehydrated Fe^II^,Fe^III^-greenalite.

Therefore, the silicates greenalite, minnesotaite, cronstedtite, riebeckite and stilpnomelane may form following the hydrolysis/oxidation of ferrous iron in anoxic alkaline high-subcritical water and contain the ferric iron Fe^III^.

#### Formation of Carbonates

The diagram drawn for the activity of Fe^2+^ as a function of pH (Fig.14 in Eugster and Chou 1973) shows that the solubility of siderite is very low at pH 9 to 14 at 25 °C. Bénézeth et al. (2009) studying the effect of temperature at pH ~6, showed a decrease of the solubility product of siderite from 25 °C to 250 °C.

Therefore, it appears that siderite precipitates when T increases and at alkaline pH. It is possible to advance that at hsc water and high pH, siderite forms and precipitates easily. The formation of siderite trough the hydrolysis of fayalite is an exothermic process (Bassez 2017a, b) which does not depend on pH and T as much as the formation of Fe^III^. Therefore, long lengths of siderite without ferric oxides neither silicates may be observed.

#### Dissolution of Silica

The solubilities of crystalline and amorphous silica in water have been widely experimentally and theoretically studied. Diagrams show that both solubilities increase with pH, and T&P apart from an exception at the critical point of water, as described below:

##### *Solubility of Amorphous Silica Versus Quartz*

Fournier and Rowe (1977) show that amorphous silica dissolves in greater amount than quartz from 25 °C up to the critical point of water. Williams and Crerar (1985) report the same trend in their Fig.1b (upon Walther and Helgeson 1977) with a difference in solubilities which seems to decrease at 350 °C. Karasek et al. (2013a) show in their Fig.4 that the solubility ratio of amorphous silica to quartz decreases from ~18 at 25 °C to ~2 at 380 °C.

**Fig. 1:**
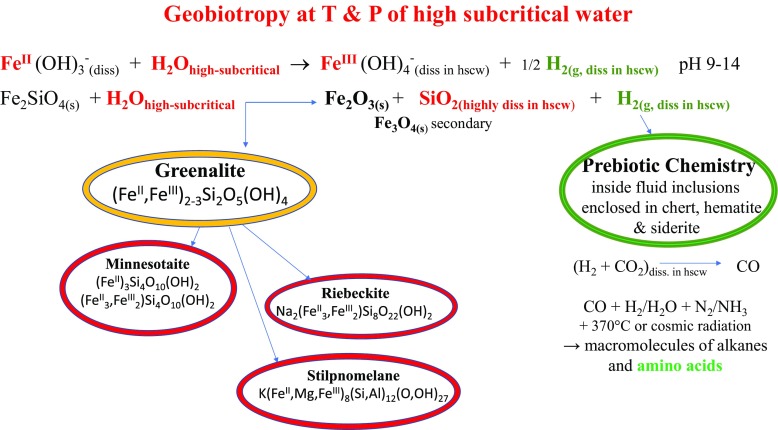
The process of geobiotropy in anoxic alkaline high-subcritical water, showing the oxidation of ferrous iron into ferric iron, the hydrolysis of fayalite connected to the high dissolution of silica, and the release of H_2_. Hematite is a primary product, while magnetite is secondary. Fe^III^-greenalite and its dehydrated Fe^III^-silicates are produced when water is super-saturated in SiO_2_. Prebiotic chemistry is triggered by the release of H_2_ and the formation of CO in T&P conditions of high-subcritical water. Diss = dissolved, hscw = high-subcritical water

Therefore, I conclude that at high-subcritical conditions of water SiO_2_ dissolves in about equal amount of amorphous and crystalline quartz and that with decreasing temperature, a larger amount of dissolved SiO_2_ stays in the amorphous state.

With the perspective of the scenario developed for the formation of BIFs in the next chapter, it seems interesting to consider here an earlier study at 25 °C on the progressive diagenesis of silica deposits during burial (Williams et al. 1985 and refs herein). They consider silica deposits as an *“amorphous silica (opal-A)”* phase which transforms upon the diagenetic sequence opal-A → opal-CT → quartz. Their Fig.6 shows the progression of diagenetic zones in a sedimentary-rock stratigraphic column. First, deposits of diatom and radiolarian tests show amorphous (opal-A) silica polymorphs which are metastable and transform upon burial: *“a less soluble disordered cristobalite-tridymite phase (opal-CT) forms…The opal-CT then recrystallizes to still less soluble quartz…Similar textures are observed in the opal-A*→ *opal-CT transformation in petrified wood (Stein* 1982*), and in hydrothermal studies of amorphous silica, silica sols, and gels…”* Williams et al. describe the aqueous solubility of the three silica species paralleling the diagenetic sequence with at 25 °C, ~60–130 ppm for amorphous silica, ~20–30 ppm for cristobalite and ~6–10 ppm for quartz, and they describe the relative dissolution rates following the same order at 25 °C and pH 8.5. This 1985 description of higher solubility of amorphous silica than quartz at 25 °C is confirmed in the above 2013a description of Karasek et al.

##### *Solubilities of Amorphous Silica and Quartz as a Function of T&P*

**Fig. 2:**
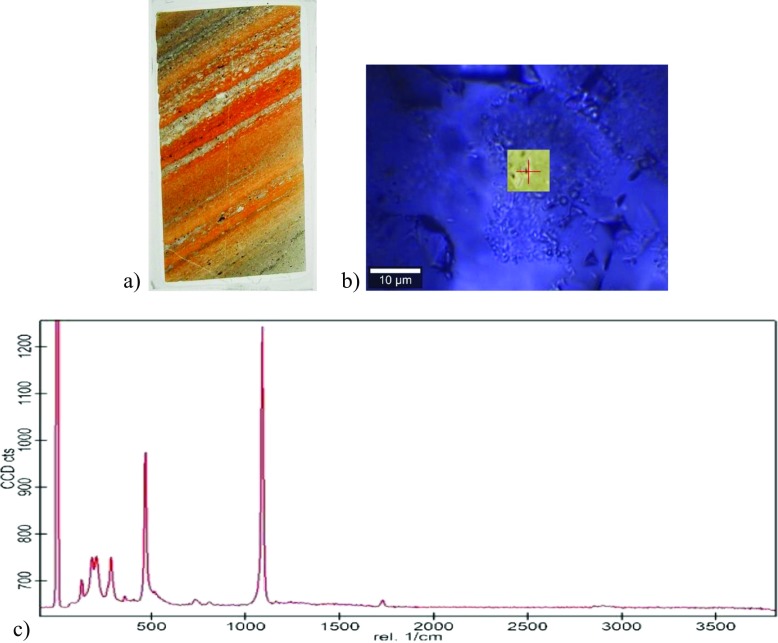
**a** The polished thin section 23B (2 cm × 4 cm × 30 μm) of a sample from the BARB3 drill core through Buck Reef Chert (easting 36 J 292202.67 northing 7,130,634.87; i.e. 25° 55.685’ S 30° 55.511′ E) **b**) Image in Transmitted Light with the Witec alpha300 confocal microscope equipped with the objective 100× **c**) Raman spectrum taken with the Witec spectrograph at the location of the red cross in b; the broad scan is between −80 cm^-1^ et +3800 cm^-1^; laser light: 532 nm

Fournier and Rowe (1977) and Williams and Crerar (1985) in their Fig.1b show that both solubilities increase with T with a turn-over at water Tc and that they increase also with P. With the 3-D diagram of Fig.3 in Karasek et al. (2013a) it appears clearly that the aqueous solubility of amorphous silica increases continuously with T&P with a turn-over at the critical point of water. This discontinuity in solubility was already reported in several earlier articles. For instance, the solubility of quartz in neutral water was calculated up to 800 °C and 2000 MPa (Smith Jr and Fang 2011). Their Fig.2 shows an abrupt change at the critical point of water and below 100 MPa. *“At a constant pressure of 23 MPa, the SiO*_*2*_ *solubility increases to a value of 0.087 wt% at 350 °C and drastically decreases to 0.0081 wt% at 450 °C*. This behavior was already specified by Shock et al. (Fig. 14, 1989) and Manning (Fig. 6, Manning 1994), through compilations of experimental results. The abrupt change can probably be explained by the change in the dielectric constant of supercritical water (Liebscher 2010 and refs herein) and the fact that non-polar supercritical water contains non-polar water dimers (Bassez 2003) which do no dissolve ion species.

This dissolution of silica in high-subcritical water is confirmed in an experiment conducted on fused silica capillaries, FSC, of 7 cm long, 500 μm internal diameter, exposed to supercritical water, scw, (Karasek et al. 2013b). At 40 MPa, during an increase of temperature between 250 °C and 400 °C, the external “*surface becomes progressively more coarse-grained indicating the increasing ability of water to corrode the silica surface*”, while “*a 4-h treatment with stagnant SCW at 400°C and 400 bar did not produce any apparent effect on the inner surface.”* Thus, I conclude that in high-subcritical water, silica dissolves and in low supercritical water silica does not dissolve.

Therefore, the solubilities of amorphous and crystalline silica increase with T and P from 25 °C up to the critical point where a drastic decrease operates. They are high in high-subcritical water and decrease abruptly in supercritical water, below 100 MPa.

##### *Solubilities of Amorphous Silica and Quartz as a Function of pH*

The solubility of silica as a function of pH is also studied. Fig.1a in Williams and Crerar (1985) after Volosov et al. (1972) shows at 25 °C for pH <~9, the monomeric silicic weak acid H_4_SiO_4_, which is the predominant aqueous species in undersaturated silica solutions, for pH ~9–13 the H_3_SiO_4_^-^ species and for pH >~13 the H_2_SiO_4_^2-^ species. This diagram indicates a drastic increase in the silica solubility above pH 9. Also, at pH >~9 the polymeric species Si_4_O_6_(OH)_6_^2-^ forms (Williams et al. 1985 and refs herein; Baes Jr and Mesmer 1976).

When dry amorphous silica is dissolved at 250 °C–300 °C in a Parr minireactor and quenched at 70 °C, nanoscale α-quartz precipitate with an average size of 18 nm. Same results were obtained with fumed silica and amorphous colloidal silica. The value of pH was maintained at ~8 because *“more basic solutions will favor the dissolution of solid phases of silica”…“Lower temperatures and more neutral pH conditions result in unreacted amorphous product, the formation of cristobalite, or complex silicate phases…Quenched products contain significant fractions of amorphous silica.”* (Bertone et al. 2003 and refs herein).

Therefore, as the solution of silica (SiO_2_) in polar water becomes more alkaline, the solubility of silica increases, and when amorphous silica is dissolved at 250 °C–300 °C and then quenched at 70 °C, α-quartz precipitates with incorporation of amorphous silica.

##### *Conclusion on the Mineral Syntheses*

In the scenario for the formation of BIFs, upon the experiments described in the above paragraphs on *Solubilities of Amorphous Silica and Quartz as a function of T&P,* amorphous silica and quartz dissolve in hsc water and not in low supercritical water. Considering the studies reported in the paragraphs on the *Solubility of Amorphous Silica* versus *Quartz,* the silica which is dissolved in hsc water, interacts with the ocean cool water in a quenching process, and is distributed in equal amounts of amorphous and crystalline quartz. The amorphous silica transforms further on, upon the diagenetic sequence opal-A → opal-CT → quartz, depending on the burial depth and the interaction with meteoric water, until the formation of α-quartz. This crystalline form of quartz can differ from the crystalline quartz which formed in hsc water, thus leading to two different quartz grains in BIFs.

However, upon the Bertone experiment described in the paragraphs on *Solubilities of Amorphous Silica and Quartz as a function of pH*, the quenching process in the cool ocean waters which can be around 70 °C, should lead to nanoscale α-quartz with an amount of amorphous silica which depends on the solution pH, more neutral pH producing more amorphous silica.

As a consequence of these paragraphs on silica, in alkaline high-subcritical water, silica-undersaturated water should trigger the hydrolysis and carbonation of fayalite/ferrosilite leading to the described minerals.

#### Related Laboratory Experiments

Equations of Table 1 are proven in some laboratory experiments that were reported in Bassez 2017b. Below are reported other experiments connected to these equations. For instance, a very recent article (Alonso-Domínguez et al. 2017) demonstrates in a laboratory experiment analyzed with X-ray diffraction, XRD, that the nanoparticulated spinel-type iron oxides magnetite and maghemite γ-Fe_2_O_3_ crystallize in supercritical water at 450 °C and 25 MPa, while oxide hydroxides and hematite α-Fe_2_O_3_ crystallize at lower temperature 200 °C and 380 °C. This experimental result is in agreement with the differential scanning calorimetry, DSC, experiment conducted up to 685 °C (Dekkers 1990), where natural goethite, with ~13% H_2_O attached to FeO(OH) and no titanomagnetite neither pyrrhotite but perhaps trace amounts of organic matter, converts into hematite between 260 °C and 360 °C with trace amounts of magnetite above 400 °C. *“At 685°C, the magnetic mineralogy was usually dominated by magnetite*”. Thus, it appears that goethite and hematite form at high-subcritical conditions of water and magnetite at 400 °C and above.

Equation 7 in Table 1 can also be recognized in an experiment, conducted on the dissolution of natural occuring fayalitic olivine (Qafoku et al. 2012). The presence in the unreacted olivine of the amphiboles cummingtonite (Mg,Fe,Mn)_7_Si_8_O_22_(OH)_2_ and protoferro-anthophyllite (Fe,Mn,Mg)_2_Fe_5_Si_8_O_22_(OH)_2_ is identified by X-ray diffraction. And it is reported that these two amphiboles were *“unreactive under all experimental conditions”.* The transmission Mössbauer spectrum shows ~90% of the total Fe associated with fayalite, ~3% with magnetite and ~7% with Fe^III^-oxide. The dissolution of fayalite was studied up to 85 days at 35 °C, 50 °C, 80 °C, 9 MPa and anoxic conditions, both in a two-phases liquid water scCO_2_ system, and in H_*2*_O-saturated scCO_2_. The supercritical state of CO_2_ starts at Tc = 31.1 °C and Pc = 7.38 MPa. At all temperatures, in the presence of liquid water and scCO_2_, SEM images of the reacted fayalite show 2–10 μm rhombohedral crystals attributed to siderite ((Fig.3abc in Qafoku et al. 2012). *“The lack of any clear relationship between the particle morphology and the fayalite surface indicates non-epitaxial growth of siderite...and growth in solution possible.”*

The particles that I observe on the SEM images of the 7-days reacted fayalite at 80 °C in liquid water scCO_2_ (Fig.3a in Qafoku et al. 2012) appear bigger and more regular (5–10 μm) than those observed on the SEM images of the 7-days reacted fayalite at 80 °C in H_2_O-saturated scCO_2_ (Fig.4 in Qafoku et al. 2012). The Mössbauer analysis of these two types of reacted fayalite shows identical spectra, fitted with 3% siderite. Thus, the synthesis of well-defined crystals of siderite appears to require a liquid water phase. This process of carbonation seems to start at temperature as low as 35 °C at 9 MPa in the presence of water. This formation complies with Eq. (7) which is calculated exothermic and spontaneous at low-T in Bassez (2013–2017). It shows also the necessity of the interconnected equations of hydrolyses and carbonations of Table 1 since CO_2_ needs to be in contact with liquid water.

When the 80 °C dissolution of fayalite in H_2_O-saturated scCO_2_, is extended to 43 days, the SEM images of the fayalite surface show ~500 nm needle-like precipitates in platelet-like morphology (Fig.6 in Qafoku et al. 2012). TEM analysis concluded in a hematite nanocrystal core covered with a silica-rich layer. *“An increase in reddish appearance”* is observed. TEM-selected area electron diffraction, TEM-SAED, analysis conducted underneath the hematite platelets show two separate crystalline phases of siderite and fayalite with no crystallographic relationship and ~10 to 20 μm siderite vertical growths composed of rythmic horizontal layers with a 3 to 4 μm *“rhombus morphology at the base”.* As mentionned by Qafoku et al. 2012, *“formation of water film, necessary for siderite growth to occur, has been reported to take place at nanometer thickness at the forsterite surface under H*_*2*_*O-saturated scCO*_*2*_ *environments* (Loring et al. 2012). *We speculate that such film formation can also occur on fayalite...”.*

Thus, in experimental conditions far from the critical point of water, at 80 °C and 9 MPa, and in H_2_O-saturated scCO_2_, in other words in supercritical CO_2_ containing a small amount of water, fayalite seems to dissolve very slightly on a nanometer scale, forming a lower layer of hematite overlained by silica. It is to be noticed that the hematite and Fe^III^-silicates synthesis, as in Table 1, require high-subcritical conditions of water, at least 300 °C and 10 MPa. The Qafoku et al. experiment if conducted at least at 300 °C and 10 MPa, would most probably show a greater amount of hematite. Magnetite is not observed since it should appear above 400 °C (see above) and siderite is observed complying the same exothermic reaction (7) of Table 1, than in the 7-days experiment when liquid water is in contact with scCO_2_.

Another experiment was conducted on dissolution of natural Mg-rich olivine crystals, extracted from a non-serpentinized peridotite (Olsson et al. 2012). After 4 days of reaction with oxygen deficient water at 120 °C and 5.5–6.5 MPa, in subcritical CO_2_, the produced crystals were covered with a red product (Figs 8 to 10 in Olsson et al. 2012). XRD pattern “*matches forsterite, hematite and magnesian calcite.”* X-ray photoelectron spectroscopy, XPS, shows a peak full width at half maximum, FWHM, which slightly extends towards higher binding energies suggesting *“the formation of carbonates minerals, clays or silica”.* SEM images of the reacted olivine, show individual 1–2μm long, ~100 nm thick, needle-like crystals associated in clusters (~800nm) “*characteristic of goethite (Cornell & Schwertmann* 2003*)*” as reported by Olsson et al. (2012). Thus, at 120 °C, ~6 MPa, far from the supercritical point of water, goethite or hematite seems present and magnetite is not. As Olsson et al. write: *“What is remarkable in our experiments is that we consistently produce hematite and not the usual magnetite”.*

Upon comparison of the SEM images of Fig.10b in Olsson et al. (2012) and Fig.6b in Qafoku et al. (2012), I advance that the hematite particles of the Olsson Mg-rich olivine experiment morphologically approach those observed in the Qafoku experiment on natural fayalite/olivine dissolution and assigned to hematite covered with silica. Thus, both both the Mg-rich olivine (Olsson et al. 2012) and natural occuring fayalitic olivine (Qafoku et al. 2012) experiments show small amounts of hematite at lower T&P than high-subcritical conditions of water, which is unexpected by Eq. (1) of Table 1 of the present article. But they show also no magnetite as expected. The broadening of the X-ray Photoelectron Spectroscopy, XPS, spectrum towards high-binding energies (Fig. 9 in Olsson et al. 2012) may originate in the high silica amount which is released by all hydrolysis and carbonation reactions, as shown by Bassez since 2013.

Another experiment shows that magnetite is not observed or only in trace amounts when lherzolite peridotite is altered by artificial seawater at 200 °C & 50 MPa, pH 12.1. The experiment was conducted by Seyfried Jr et al. (2007) and cited in Bassez (2013–2017). In extra, the presence of ferric iron is observed in two Mössbauer doublets indicating the presence of both Fe^3+^ and Fe^2+^ in the octahedral layer of the serpentine and thus confirming my above proposition of incorporation of Fe^III^ inside the silicates which are the products of the hydrolysis of Fe^II^-silicates by alkaline hsc water (section on *Formation of Fe*^*III*^*-silcates*).

Another experiment shows that magnetite forms only with increasing temperature: San Carlos olivine was altered at 250 °C–350 °C, 50 MPa. It is observed that: *“iron was preferentially incorporated into magnetite with increasing temperature and was exclusively hosted by magnetite at 350°C “* (Malvoisin et al. 2012).

Finally, other experiments conducted at 300 °C, 35 MPa, on harzburgite containing pentlandite and pyrrhotite (Klein et al. 2015; Grozeva et al. 2017), show that magnetite appears associated with the sulfides.

Synthetic fluid inclusions in olivine allow to follow serpentinization progress at 280 °C and 50 MPa (Lamadrid et al. 2017). The description of the experiment shows that crystals of brucite and serpentine start to appear after 15 days and magnetite after 120 days. Table 1 in Lamadrid et al. (2017) shows that magnetite is not observed in the analysis with the sample at 280 °C when the sample is removed from the furnace and quenched to room temperature for Raman analysis. That would mean that magnetite is transformed quickly with temperature and that, for conclusions on the presence of magnetite, it is important to keep the temperature of the reaction during the analysis.

As a conclusion on the analysis of these *Related Laboratory Experiments*, the peridotite silicates dissolve slightly in anoxic supercritical CO_2_. Hematite starts to form in very low amount and nanoscale sizes at low subcritical conditions of water (80 °C & 90 bar). Magnetite seems to form mainly in low supercritical water. When sulfides are present in the peridotites, magnetite seems associated with the sulfides possibly following the endothermic equation of hydrolysis of sulfides instead of silicates, as proposed in Bassez (Bassez 2013–Bassez 2017a, b).

11 $$ \mathrm{FeS}+4/3{\mathrm{H}}_2\mathrm{O}\to 1/3{\mathrm{Fe}}_3{\mathrm{O}}_4+{\mathrm{H}}_2\mathrm{S}+1/3{\mathrm{H}}_2\kern1em {250}^{{}^{\circ}}\mathrm{C},\kern0.5em \mathrm{pH}\ 3.5-8 $$

#### Conclusion of the Chapter on the Formation of Ferric Minerals in Anoxic Water

The high solubility of SiO_2_ at high-subcritical, hsc, conditions of water compared to its extremely low solubility just above the critical point, triggers the dissolution of ferrous silicates with production of quartz and amorphous silica in equal amounts. The ferric oxide hydroxides goethite and lepidocrocite and their dehydrated product hematite form in the basic hsc water, together with Fe^III^-greenalite. Silica is adsorbed on goethite and hematite, inducing more dissolution of the ferrous silicates. The solution becomes progressively supersaturated in SiO_2_, in a process which allows the formation of the dehydrated products minnesotaite, and most probably riebeckite and stilpnomelane. Minnesotaite, riebeckite and stilpnomelane can also incorporate the ferric iron Fe^III^. Siderite is produced in an exothermic reaction which can proceed at various T&P conditions (Bassez 2013–2017).

When laboratory hydrolysis occurs near the critical point of water (200 °C, 50 MPa), ferric iron is experimentally observed incorporated inside the silicate products. Thus, the new Eq. 1 of Table 1 of the present article, which describes the production of Fe^III^ iron in anoxic and abiogenic conditions at high-subcritical conditions of water and high pH, can explain the incorporation of Fe^III^ inside oxides and silicates, in the absence of oxygen and microorganisms. This conclusion is applied in the next chapter to propose an alternative model for the formation of the minerals in Archean banded iron formations. As a conclusion of the present chapter, it appears that values of high pH, and T&P for high-subcritical water are determinant for the production of Fe^III^, silica in amorphous and crystalline phases, ferric oxides and ferric silicates, in anoxic and abiogenic conditions.

### High-Subcritical Anoxic Alkaline Water for the Formation of the Minerals Observed in Banded Iron Formations

In this chapter, I show that the anoxic and abiogenic formation of ferric iron in high alkaline and high-subcritical water can contribute to elucidate the chemical composition of Banded Iron Formations, BIFs. Indeed, the minerals described in the precedent chapter as products of the hydrolyses/oxidations and carbonations of fayalite/ferrosilite, and of the consecutive dehydrations, are those observed in BIFs.

#### Reported Mineralogies of BIFs

For instance, BIFs are observed in the ~2.5 Ga Hamersley Group of the Pilbara craton, Western Australia and the Transvaal Supergroup of the Kaapvaal craton, South Africa. These formations are well-documented (Beukes 1984, Rasmussen et al. 2017, Haugaard et al. 2016, and refs herein) and correlation diagrams from late Neoarchean to early Paleoproterozoic stratigraphic successions, observed in the Hamersley and Transvaal basins, are drawn in Figs.5 & 6 in Beukes and Gutzmer 2008.

In 1984, N.J. Beukes observed the iron formations of Griquatown and Kuruman both in the Asbesheuwels Subgroup of the Ghaap group, in the Griqualand west basin of the Transvaal Supergroup that overlies the Kaapvaal craton in South Africa and writes that they are *“correlatives of the Penge iron formation...unmetamorphosed and structurally little deformed*…*oxidized to jasperoids down to depths of several tens of metres...All mineralogical information is...from diamond drill core”.* The minerals siderite, greenalite, minnesotaite, riebeckite are described in the Danielskuil, Skietfontein and Middelwater members of Griquatown and siderite, hematite, magnetite, stilpnomelane, chert in the Groenwater member of Kuruman. N.J. Beukes suggested *“a link between the deposition of stilpnomelane and silica”* and noticed that the stilpnomelane which is described by Trendall and Blockley (1970) in the Dales Gorge member of the Australian Brockman iron formation, is equivalent to the stilpnomelane observed in the South African Kuruman formation.

A very recent article reports high-resolution microscopy observations connected to energy dispersive X-ray spectroscopy, EDS, and selected area electron diffraction, SAED, of μm and nm iron-silicate particles in samples from the Griqualand West BIF succession (Rasmussen et al. 2017 and refs herein). Three formations are studied: Kuruman, Klein Naute and upper Nauga.

- Chert of the 222.30 m deep Klein Naute formation (Fig. 6&7 in Rasmussen et al. 2017) comprises interlocking 5 to 50 μm quartz grains and 50 nm to 1 μm nanoparticles of iron silicate *“comparable to greenalite nanoparticles from the Hamersley Group*”. In about 5% of the cherts, densely packed greenalite form up to 50 μm euhedral polygons surrounded with μm-sized siderite crystals. Alternated 0.2 to 0.4 mm upper layers of polygons lie on microlaminated bases lined with siderite or ankerite-dolomite crystals.
- In the 327.11 m upper Nauga formation (Figs. 9 and 10 in Rasmussen et al. 2017) individual particles of iron silicate are observed with sizes from 50 nm to 1 μm. The SAED patterns and the high-resolution electron microscope HREM measurements *“closely match the mineral greenalite”*. Alternating layers of densely packed iron-silicate nanoparticles and siderite or ankerite-dolomite crystals are also observed as in the Klein Naute formation (Fig.7A) and in the 326.69 m upper Nauga (Fig.14).
- The laminated chert layers with iron silicate particles in the 356.54 m upper Nauga formation (Fig.12 A-C) contain 0.2–0.5 mm, fan-shaped crystals of minnesotaite *“replacing chert and silicate nanoparticles”.* The minnesotaite has “*destroyed lamination and polygonal structures”.*
- In the nodular chert layers of the 183.16 m Kuruman formation, banded magnetite (at least 1 cm thick and 3 cm long) (Fig. 12D) alternates with nodular chert which appears near completely replaced by a mineral identified as minnesotaite (Fig. 12 F&G).

Therefore, greenalite appears associated with siderite (a&b) and minnesotaite appears replacing silica and silicate nanoparticles (most probably greenalite) in (c) and silica in (d). The minerals greenalite and siderite can be the products of primary hydrolysis and carbonation of fayalite/ferrosilite. Minnesotaite can be the product of the greenalite dehydration in water which is supersaturated with silica, thus replacing silica and greenalite. Greenalite and minnesotaite appear as primary compounds in relation to the hydrolysis and dehydration equations in alkaline hsc water that I present since 2013. The Fe-rich serpentine greenalite, and its dehydrated product minnesotaite, which are phyllosilicates, can incorporate Fe^III^ as I propose in Bassez (2016b), Bassez (2017a, b) for the Mg-rich serpentine chrysotyle which is a T-O phyllosilicate and its dehydrated product talc which is a T-O-T phyllosilicate. This Fe^III^ incorporation is confirmed in an article very recently published (Johnson et al. 2018) which analyzes samples of the ~2.5 Ga Australian and South-African BIFs on a nanometer scale and concludes that the observed low-Fe(III) greenalite is a primary mineral in BIFs. Finally, the magnetite which is observed in (d) can be a secondary product (Bassez 2013–2017) arising from thermal metamorphism above 400 °C or siderite decomposition ~450°C, 50 MPa or siderite hydrolysis at 300 °C, 50 MPa (above section on *Formation of Iron Oxides Hydroxides* in the precedent chapter).

The same chemical composition is observed in BIFs of Western Australia. The ~2.45 Ga Joffre banded BIF member of the Brockman iron formation of the Hamersley Group that overlies the Pilbara craton, has also been recently documented in detail (Haugaard et al. 2016 and refs herein). The two most dominant rock types are defined as oxide BIF and silicate-carbonate-oxide BIF. In the oxide BIF, microcrystalline chert is often found as (0.25-1 mm) microbands composed of hematite, carbonate, crocidolite (a derivate of riebeckite), alternating with microbands of magnetite and hematite. Some sections reveal (<0.01 mm) microbands of stilpnomelane. The silicate-carbonate-oxide BIF is dominated by chert, magnetite, ankerite, riebeckite, crocidolite and stilpnomelane*.* Braided network of hematite and minor goethite is occasionally observed. Alongside chert, dense micro- and meso- bands of magnetite are found as major constituent. After chert and magnetite, riebeckite is the most abundant. Two other type of rocks named the stilpnomelane-rich tuffaceous mudrock and the stilpnomelane mudrock contain microgranules of stilpnomelane.

Analyses of the sample ABDP9, drilled in the 2.63–2.45 Ga Hamersley Group, show that the 0.2 to 2 mm laminae in chert beds contain *“iron silicates (stilpnomelane, riebeckite, minnesotaite, greenalite), iron oxides (hematite, magnetite), and carbonates (ankerite, siderite, calcite, dolomite)”* Chert is composed of nanoparticles which are “*abundant, randomly oriented, elongate…ranging from <10 nm to 600 nm long and ~1nm to 50 nm wide…Transmission electron microscopy shows that they comprise iron-rich silicates: stilpnomelane and greenalite”* (Rasmussen et al. 2015).

The 3.46 Ga old Marble Bar Chert of Pilbara craton shows also the same association of minerals with chert, hematite, magnetite, siderite and greenalite (Rasmussen et al. 2014).

Therefore, the above summary of observed minerals in the ~2.5 Ga Transvaal and Hamersley BIFs and in the 3.46 Ga Marble Bar Chert are those of the anoxic hydrolyses and carbonations of fayalite/ferrosilite-rich silicate rocks together with the dehydrated products as described in Table 1 and Fig.1. Both Kaapvaal and Pilbara cratons show minerals in their BIFs which can be classified as oxides, silica, silicates and carbonates. The oxides, and silicates to a less extent, are observed to contain ferric iron. Minnesotaite, as expected for a direct dehydrated product of greenalite, appears directly associated with greenalite and chert in Transvaal. Minnesotaite is not reported, neither greenalite, in the Joffre formation (Fig.2 in Haugaard et al. 2016), where instead, the dehydrated silicates are observed, talc, riebeckite, crocidolite, stilpnomelane. The Joffre formation may be a case of high silica content of the interacting hsc water, leading to little greenalite and minnesotaite which dehydrated into stilpnomelane, riebeckite and crocidolite. Stilpnomelane is not reported by B. Rasmussen et al. (2017) in Kuruman, Klein Naute neither upper Nauga. However, it was observed by N.J. Beukes (1984) in the Groenwater member of the Kuruman formation. Stilpnomelane is observed with greenalite in the sample BDP9 of Hamersley, suggesting that stilpnomelane formed at the same time than greenalite. The nanosizes of the observed particles can originate in high-subcritical water as described in the next section.

#### A Proposed Scenario for the Formation of Minerals in BIFs

Thus, it is possible to propose that the BIF minerals of the Transvaal Supergroup and Hamersley Group, both dated late Neoarchean-early Paleoproterozoic periods, and the BIF minerals of the Archean Marble Bar Chert, formed under the same process of fayalite/ferrosilite hydrolyses and carbonation. This process, is governed first by anoxic alkaline water just below its critical point, which triggers the anoxic oxidation of Fe^II^ and consequently the formation of hematite and Fe^III^-silicates, with secondary magnetite, and second by the high dissolution of silica in high-subcritical water which leads to the dehydration products minnesotaite, riebeckite, stilpnomelane. Alkaline pH is determinant and also the T&P values of hsc water. A confirmation of the influence of hsc water is noticed in the sizes of the fine-grained and nanoparticles which are observed both in BIFs and in supercritical water laboratory experiments. These T&P subcritical conditions, that crustal silicate rocks may encounter, are probably caused by a periodic convective movement inside the mantle which is associated to a still unknown magma variation.

Therefore, the chemical Eq. (1) of Table 1, that represents the alkaline anoxic oxidation of ferrous iron into ferric iron, can be applied to explain the source of ferric iron in minerals deposits and a possible scenario may be as follows:

Fayalite is an abundant mineral which composes the rocks of the Earth’s crust and upper mantle, together with ferrosilite. During late archean times, due to hot magma (Condie et al. 2016; Condie 2016) and tectonic movement (Klein et al. 2017) numerous subduction zones create fractures leading to hydrothermal eruptions. At depth, water is much above its critical point. Above 100 MPa and 450 °C, SiO_2_ highly dissolves. Rising water decreases in temperature and pressure, and silica precipitates when at the specific T&P just above the critical point of water, as described in the section on *Dissolution of Silica* of the precedent chapter. Then, at high-subcritical conditions, part or all of the precipitated silica can redissolve. If at that point, crustal rocks containing fayalite/ferrosilite are in contact with alkaline hsc water which is undersaturated in silica, fayalite/ferrosilite can dissolve. Ferrous iron is oxidized into ferric iron with release of H_2_. The ferric oxide hydroxide goethite and the oxide hematite, form. Magnetite forms as a secondary product. It is indeed observed as crosscutting primary laminations of chert as for instance in the Moodies Group (Bontognali et al. 2013).

Silica dissolves until saturation. Upon Fig.3&4 of the Karasek et al. 2013a article, discussed in the precedent chapter, dissolved silica at high-subcritical T&P is in equal amounts of crystalline and amorphous phases. Thus, when thermal quenching occurs in cooled oceans or shallow waters, silica can keep its high T proportions and be of two types, crystalline quartz and amorphous silica glass. The quenched silica can be nanosized α-quartz containing some amorphous structures as demonstrated in the Bertone et al. laboratory experiment (Bertone et al. 2003). Depending on the pH of the hsc water, the amount of the amorphous phase varies. Since it is usually considered that the quenching process shows minerals which are produced at high T, without structural rearrangement, I propose that α-quartz forms in hsc together with α-quartz incorporating disordered domains and Fe^III^, leading to the Fe^III^-greenalite mineral. It is to be noticed that some chert of BIFs is composed of hard, very fine-grained silica. In supercritical hydrothermal synthesis conducted in the laboratory, submicron-sized particles instead of micron-sized, form, as shown in the synthesis of talc conducted in supercritical water at 500 °C and 40 MPa, followed by thermal quenching in ice (Dumas et al. 2016). Also, the Alonso-Dominguez et al. (Alonso-Domínguez et al. 2017) experiment, cited above, shows mean particle sizes around 30 nm for all the iron oxides hematite, magnetite, maghemite, and oxide hydroxides FeO(OH), which are synthesized in supercritical water. It is thus possible to propose that the silica which dissolves from fayalite/ferrosilite at conditions near supercritical, forms under sub-micrometer sizes and that the process of quenching allows these sizes to be preserved. The nanosize synthesis of minerals in near supercritical water can thus be at the origin of the sizes and textures of minerals in BIFs. They can form through fayalite/ferrosilite hydrolysis following a triggering effect produced by the alkaline interacting water in the near-supercritical state.

When water reaches saturation in SiO_2_, the dehydrated products minnesotaite forms and most probably stilpnomelane and riebeckite. However, when the rising water is silica-rich, greenalite can readily dehydrate into minnesotaite, stilpnomelane and riebeckite, leading to smaller quantities of greenalite as observed in the ~2.45 Ga Joffre banded BIF member of the Brockman iron formation, Hamersley Group (Haugaard et al. 2016, & above section). When CO_2_ is present, siderite can be produced in a hsc water which is undersaturated in silica. Considering Eqs. (2–10) it sems that neither siderite, nor silicates, nor oxide hydroxides, nor hematite can form in a rising hsc water which is already supersaturated in silica, since dissolution of fayalite or ferrosilite producing silica cannot occur.

#### Conclusion on the Formation of Minerals in BIFs

Therefore, the formation of the BIF minerals may depend on four characteristics of water which act together in a specific point of space and time at a right kairos: water in its high-subcritical state (300 °C–350 °C, 10 MPa–22 MPa), high pH (9.5–14), undersaturated in silica, and interacting with crustal rocks containing fayalite/ferrosilite. This conjunction of events in time and space may happen only rarely and depends on the temperature, pressure and composition of the rising water. Consequently, I propose that it is the state of water which is just below the supercritical state which triggers the formation of very fine-grained oxides and silicates. If water encounters fayalite/ferrosilite while in high supercritical state, containing thus high levels of dissolved SiO_2_ at saturation, no fayalite/ferrosilite dissolves. If the encounter occurs at very low subcritical conditions, where the solubility of silica is very low, no silica can dissolve and consequently no fayalite/ferrosilite dissolves. In both cases, Fe^II^ is not oxidized into Fe^III^. Hydrolysis of fayalite/ferrosilite does not start and minerals are no not ejected from a possible venting arc. After many years, hydrolysis of fayalite/ferrosilite starts again when temperature and pressure of the alkaline silica-undersaturated water reach high-subcritical values. The synthesized minerals are again ejected from plumes, creating layers depending on gravity: hematite has a specific gravity of ~5.26, magnetite ~5.18, siderite ~3.96, riebeckite ~3.30, ankerite ~3.10, minnesotaite ~3.01, dolomite~2.86, stilpnomelane ~2.59–2.96, quartz~2.59–2.65 (Anthony et al., 2004-2017). Thus, silica should overlain hematite creating adsorbed bonds as experimentally observed (section on *Dissolution of Fayalite and Formation of Dissolved Fe*^*2+*^ in the first chapter of Results). Cohesion between all molecules proceed during years. I suggest that this periodic change in temperature may result from a periodic convective process inside the mantle arising from magma variation. However, knowledge of such process is not yet enough documented.

### High-Subcritical Anoxic Alkaline Water for the Process of Geobiotropy inside Fluid Inclusions

In this chapter, I show that fluid inclusions inside rocks, may contain macromolecules of amino acids, which can be synthesized in anoxic conditions at 300 °C–350 °C or by microwave or gamma ray excitation.

#### CO Is Needed for Prebiotic Synthesis

The equation of anoxic oxidation of ferrous iron (Eq.1) releases H_2_ at ~300°C–350 °C and ~10MPa–25 MPa and not above the critical point of water. This H_2_ can form bubbles and be associated with other dissolved gases and liquids inside fluid inclusions containing for instance H_2_, H_2_O, CO_2_, N_2_, CH_4_. At these specified temperatures and pressures, hydrogenation of carbon dioxide can proceed to form CO. It is worth here remembering the 1902 Sabatier & Senderens reactions which showed direct formation of methane from mixtures of gaseous H_2_ and CO_2_ at 350 °C with Ni, and 300 °C (with higher rates at 360 °C and 400 °C) with Co. Methane did not form with Cu at 430 °C, instead CO formed, and neither CH_4_ nor CO formed below 420 °C with Fe (Sabatier and Senderens 1902). A more recent experiment conducted with H_2(g)_ at 500 °C on CO_2(g)_ with the catalyst Cu/Al_2_O_3_, produced CO and not CH_4_ (Chen et al. 2000), thus confirming the Sabatier reaction. Fischer-Tropsch reactions (1923) were involved in the transformation of gaseous CO&H_2_ into liquid hydrocarbons at 450 °C-50 bar in the presence of Fe. More recent experiments conducted on mixtures of dissolved H_2_ and CO_2_ in sea water at 250 °C–300 °C, 25 MPa and Fe_3_O_4_ as catalyst, showed formation of dissolved CO with minor dissolved CH_4_ (Fu and Seyfried Jr 2009)_._ The Fu & Seyfried conditions are those of hsc water.

12 $$ {\mathrm{CO}}_{2\left(\mathrm{dis}\right)}+{\mathrm{H}}_{2\left(\mathrm{dis}\right)}\to {\mathrm{CO}}_{\left(\mathrm{dis}\right)}+{\mathrm{CH}}_{4\left(\mathrm{dis}\right)}\kern1em {250}^{{}^{\circ}}\mathrm{C}-{300}^{{}^{\circ}}\mathrm{C},25\ \mathrm{MPa},{\mathrm{Fe}}_3{\mathrm{O}}_4 $$

(Fu and Seyfried Jr 2009)

When CO_2_, H_2_, CO and CH_4_ are dissolved in the aqueous liquid solution of a fluid inclusion, they coexist with their vapor phases. Therefore, following the formation of CO, either in gaz or dissolved phases, prebiotic chemistry based on CO and N_2_ in the gas phase, can proceed either with heat through Sabatier-Senderens/Fischer-Tropsch reactions (CO + H_2_) associated to Haber-Bosch (H_2_ + N_2_) reactions, or with active nitrogen (N_2_*) produced in microwave excitation, or with gamma-ray excitation as described below.

#### Published Laboratory Experiments of Prebiotic Synthesis (Table 2)

In 1996, Nils Holm proposed that Fischer-Tropsch Type synthesis of organic compounds could occur following the serpentinization of peridotite in the oceanic crust. In 2003 Hill & Nuth conducted a Sabatier-Senderens/Fischer-Tropsch & Haber-Bosch experiment with a gas mixture of 75 Torr CO, 75 Torr N_2_ and 550–650 Torr H_2_ (~1 atm pressure) and temperatures between 200 °C and 600 °C. The catalysts are iron silicates smokes containing mainly iron silicates and silica and minor iron oxides Fe_2_O_3_ and Fe_3_O_4_. “*The individual grains are nanodimensioned (~20-50 nm) and have enormous defect-rich surface areas that provide numerous sites for catalysis.”* Figures concerning experiments at 300 °C and 400 °C during ~70h, report the presence of nitrogen-containing organic molecules, “*C-N species*” assigned to methyl amine CH_3_NH_2_, acetonitrile CH_3_CN and *N*-methyl methylene imine CH_3_N=CH_2_.

**Table 2:** Amino-acids precursors (Hill&Nuth) and amino-acids formed during the combined Sabatier-Senderens/Fischer-Tropsch & Haber-Bosch reactions (left column) and during γ-ray/proton excitation (right column)

Sabatier-Senderens /Fischer-Tropsch & Haber-Bosch	Compounds names	Proton/gamma excitation
Hill and Nuth 2003 300 °C, N_2_, H_2_, CO		
CH_3_NH_2_	methyl amine	
CH_3_C≡N	acetonitrile, methylcyanide	
CH_3_N=CH_2_	*N*-methyl methylene imine	
Pizzarello 2012, 370 °C, NH_3_, H_2_, CO		Bassez et al. 2012 Excitation by proton of N_2_, H_2_O, CO Racemic amino acids are observed
NH_2_CH_2_COOH	glycine, Gly	NH_2_CH_2_COOH
CH_3_CH(NH_2_)COOH	alanine, α-alanine, Ala 2-amino propanoic acid	D,L-CH_3_CH(NH_2_)COOH
CH_3_CH_2_CH(NH_2_)COOH	2-amino butyric acid α-amino butyric acid, α-ABA 2-amino butanoic acid	D,L-CH_3_CH_2_CH(NH_2_)COOH
CH_3_C(CH_3_)(NH_2_)COOH	2-amino isobutyric acid, Aib, 2-methylalanine 2-amino 2-methylpropanoic ac.	
CH_3_CH_2_CH_2_CH(NH_2_)COOH	norvaline 2-amino pentanoic acid	
CH_3_(CH_2_)_3_CH(NH_2_)COOH	norleucine 2-amino hexanoic acid	
CH_3_CH_2_C(CH_3_)(NH_2_)COOH	isovaline, Iva 2-ethylalanine 2-amino 2-methylbutanoic acid	
	aspartic acid, Asp 2-amino butanedioic acid	D,L-HOOCCH_2_CH(NH_2_)COOH
	β-alanine, β-Ala 3-amino propanoic acid	NH_2_CH_2_CH_2_COOH
	serine, Ser 2-amino 3-hydroxy propanoic ac	D,L-HOCH_2_CH(NH_2_)COOH

The 2012 Pizzarello experiment was conducted with CO, H_2_, and NH_3_ gases in 1:1:1 ratio at 370 °C for 24 h, and with meteoritic minerals as catalysts. “The tubes were evacuated” and pressure of the reaction was a few mm Hg (Pizzarello, personal communication 2017). Amino acids (in nmol/g) were detected: glycine (436) and alanine (407) were obtained with the iron meteorite Santiago Papasquiaro (7.48% Ni). The magnetite powder led to 2-amino isobutyric acid (540.0), glycine (121.9), alanine (88.0), 2-amino butyric acid (20.3), norvaline (4.5), norleucine (2.2), isovaline (1.0).

The following paragraph introduces the characteristics of the detected amino-acids together with their nomenclature. Glycine NH_2_CH_2_COOH and alanine CH_3_CH(NH_2_)COOH are protein α-amino carboxylic acids (with group -COOH) coded by human DNA. The Aib, 2-amino isobutyric acid, C^3^H_3_C^2^(CH_3_)(NH_2_)C^1^OOH is also named 2-amino 2-methylpropionic acid and 2-methylalanine. Iva, Isovaline, C^4^H_3_C^3^H_2_C^2^(CH_3_)(NH_2_)C^1^OOH, an isomer of the human DNA-coded valine, is also named 2-amino 2-methylbutanoic acid and 2-ethylalanine. The two α-amino acids, Aib and Iva, are not coded by human DNA, but are synthesized by fungi. A GC/SIMS-MS on Chirasil-_L_ Val*,* culture*-*analysis of 49 species of fungi, show that Aib is of higher abundance and Iva forms in (*R*) (=D) configuration or as a mixture of (*S*) (=L) and (*R*) (=D) enantiomers (Brückner et al. 2009). The non-human-DNA-coded 2-amino butyric acid, C^4^H_3_C^3^H_2_C^2^H(NH_2_)C^1^OOH, also named 2-amino butanoic acid and α-amino butanoic acid, α-ABA, is found in the exudate of germinating canola seeds (Moe 2013). Norvaline CH_3_CH_2_CH_2_CH(NH_2_)COOH, named also 2-amino pentanoic acid and norleucine CH_3_(CH_2_)_3_CH(NH_2_)COOH, named also 2-amino hexanoic acid are synthesized by the bacterium *Escherichia coli* (Biermann et al. 2013). The non-human-DNA coded isovaline and norvaline are observed in racemic amounts in the Murchison meteorite together with the extraterrestrial 2-amino-2,3-dimethylpentanoic acid, 2-a-2,3-dmpa, CH_3_CH_2_CH(CH_3_)C(CH_3_)(NH_2_)COOH, which is detected with L-enantiomer excess, L_ee_ (Cronin and Pizzarello 1997). Another analysis on different samples of the Murchison meteorite shows a large L_ee_ of isovaline, norvaline and valine (Glavin and Dworkin 2009). Some of these amino acids are described in (Bassez 1998-2018).

Active nitrogen can also excite CH_4_ or mixtures of CO + H_2_, in the gas and liquid phases. Active nitrogen is composed of nitrogen atoms in the ground state N(^4^S) and nitrogen molecules in the ground singlet state N_2_ (X^1^Σ_g_^+^) and in the excited triplet state N_2_ (A^3^Σ_u_^+^). The transition A^3^Σ_u_^+^ − X^1^Σ_g_^+^ being forbidden, active nitrogen shows a high reactivity (Bassez 1971). Active nitrogen formed with microwave radiation, at ~2.45 GHz from a magnetron, in a reaction chamber at a few mmHg and was used to study the diatomic radicals NS, NSe and NTe (Bassez 1971). The formation of cyanides was reported by M. Berthelot (1868) when “*free nitrogen*” reacted with acetylene C_2_H_2_ in spark discharges. Blades and Winkler (1951) found that nitrogen atoms react on methane CH_4_ and ethane C_2_H_6_ leading to hydrogen cyanide HCN at temperatures between 350 °C–450 °C, and not at 200 °C. Moser et al. (1968) reported a peptide synthesis through HCN polymerization in alkaline medium as described in (Rauchfuss 2008 p.104). Cyanides being very reactive it is most probable that molecules of biological interest form when CH_4_ or light hydrocarbons or mixtures of CO_2_ and H_2_/H_2_O in anoxic alkaline high-subcritical water are excited with active nitrogen.

Another kind of synthesis of amino acids occurs when mixtures of simple molecules including CO are excited by particles of cosmic radiation, as demonstrated in the Kobayashi experiments (Kobayashi et al. 1990–Kobayashi et al. 2008). Macromolecules composed of amino-acids form when mixtures of gaseous CO, N_2_, above liquid H_2_O or CO, NH_3_, H_2_O, or some of the various elementary molecules CH_4_, CO_2_, CO, N_2_, NH_3_, H_2_O, are excited with particles of matter and interaction of the cosmic radiation such as, protons, He ions, e^-^, soft X-rays, γ-rays. The *G*-value of glycine, which is the number of molecules formed per 100 eV absorbed, is 0.02 for gamma rays of low-dose rate (<5 Gy/h or 5 J.kg^-1^.h^-1^). This *G*-value is the same with 3 MeV proton beams. The *G*-value is much lower, 0.001, for gamma rays of high-dose rate (>90 Gy/h) (Kobayashi et al. 2008). The yellow-brown products obtained with 3 MeV proton irradiation of (CO, N_2_, H_2_O) when analyzed by Gas Chromatography-Mass Spectrum, GC-MS, shows in decreasing amounts, glycine and racemic mixtures of D and L enantiomers: D,L-alanine, (D,L-Ala); D,L-α-amino butyric acid (D,L-α-ABA); D,L-aspartic acid (D,L-Asp); β-alanine (β-Ala); D,L-serine (D,L-Ser) and others in minor amounts (Bassez et al. 2012). β-alanine NH_2_CH_2_CH_2_COOH is achiral. Lower resolution chromatograms conducted earlier showed approximately the same amino acids: glycine, D,L-alanine, β-alanine, D,L-aspartic acid, L-serine HOCH_2_CH(NH_2_)COOH (Kobayashi et al. 1998). All of the above described amino acids, products of Sabatier-Senderens/Fischer-Tropsch & Haber-Bosch and gamma-excitation reactions are summarized in Table 2.

These abiotic syntheses of amino-acids require CO to form and not CO_2_. This fact was observed by Schlesinger and Miller (1983) and Kobayashi et al. (1990). I propose that CO can form inside fluid inclusions when CO_2_ and H_2_ are present at ~300 °C–350 °C and 10~25 MPa. These T&P values are those of high-subcritical water which allow silica to dissolve and induce the hydrolysis of iron-olivine and -pyroxenes as shown in the precedent chapter. I proposed earlier (Bassez 2008) to mix peridotite, sea-water and N_2_ in reactors and then increase temperature and pressure to high-subcritical and low supercritical conditions of water. With CO_2_ included inside the rock, CO may form with H_2_ that is released during serpentinization of the peridotite, or CO_2_ can also be added to the reactor: “*De la péridotite, au contact de l’eau de mer sous HT-HP conduirait à la formation de H*_*2*_ *et CH*_*4*_ *ainsi qu’à de la magnétite. En ajoutant de l’azote liquide et/ou de la carboglace…dans un réacteur qui serait ensuite ajusté en pression et température…L’expérience pourrait également partir d’olivine et de pyroxènes…une analyse Raman permettrait l’analyse in-situ des molécules synthétisées…Les réactifs H*_*2*_*O, H*_*2*_*, CH*_*4*_*, et N*_*2*_ *qui donnent la plus grande abondance en composés organiques d’intérêt biologique dans les expériences de Miller, seraient réunis.”* In english words: *“Peridotite, in contact with sea water under high T high P would lead to the formation of H*_*2*_ *and CH*_*4*_ *and magnetite. By addition of N*_*2*_ *and/or CO*_*2*_*…in a reactor which would be adjusted in pressure and temperature…The experiment could also be conducted with olivine and pyroxenes…In situ analysis of the synthesized molecules could be conducted with Raman spectroscopy…The reactants H*_*2*_*O, H*_*2*_*, CH*_*4*_*, and N*_*2*_ *which are known to produce the highest abundance of organic compounds of biological interest in the Miller experiments, would be assembled”,* and prebiotic synthesis in hydrothermal conditions can be achieved*.* Considering the mineral part, this theoretical proposition is currently demonstrated in a very recent experiment (Lamadrid et al. 2017) which shows water of a synthetic fluid inclusion at 280 °C, 50 MPa, interacting with the olivine hosting the inclusion. The olivine seems Mg-rich. Raman spectra show serpentine, brucite, water and hydrogen. The next step of this experiment could be the introduction of CO_2_ and N_2_ or NH_3_ at 350 °C and 25 MPa inside the synthetic fluid inclusion. CO_2_ reacting with H_2_ would lead to CO and the mixture of CO, N_2_/NH_3_, H_2_ could lead to the synthesis of prebiotic molecules such as macromolecules of amino acids as described above.

#### Geological Field Observations and Discussion

Fluid inclusions are observed in many rocks (Hurai et al. 2015; Pironon et al. 2017). In 1996, D. Kelley writes: *“Analyses of fluid inclusions in plutonic rocks recovered from the slow-spreading Southwest Indian Ridge (SWIR) record CH*_*4*_ *concentrations of 15-40 times those of hydrothermal vent fluids and of basalt-hosted volcanic gases and provide the first direct sampling of CO*_*2*_*-CH*_*4*_*-H*_*2*_*O-H*_*2*_*-C bearing fluids in the oceanic crust...The inclusions may record respeciation of magmatic fluids attendant with the inward diffusion of H*_*2*_ *into the inclusions…”.*

In 2015, McDermott et al. observed a great abundance of dissolved H_2_ and CH_4_ in the ~2350m deep Von Damm Venting Field, VDVF. They propose that *“CH*_*4*_ *and the higher hydrocarbons are likely formed in H*_*2*_*-rich fluid inclusions over geological timescales…Our results indicate that CH*_*4*_*…and higher n-alkanes may form independently of actively circulating serpentinizing fluids in ultramafic-influenced systems”.*

As a complement, I like to remember here the observation I report since 2013 and that is presented in detail in Bassez (2017a, b). The Pourbaix diagram drawn by Digby D. Macdonald in (Macdonald 1992) for the system Fe-S-H_2_O shows that ferrous monosulfides are transformed at 25 °C and pH ~5.4 to 9.5, into ferrous disulfides such as pyrite, upon the well-known following equation:

13 $$ {\mathrm{Fe}}^{\mathrm{II}}{\mathrm{S}}_{\left(\mathrm{s}\right)}+{\mathrm{H}}_2{\mathrm{S}}_{\left(\mathrm{dis}\right)}\to {\mathrm{Fe}}^{\mathrm{II}}{\mathrm{S}}_{2\left(\mathrm{s}\right)}+{\mathrm{H}}_{2\left(\mathrm{g}\right)}\kern1em {25}^{{}^{\circ}}\mathrm{C},\mathrm{pH}\ 5.4-9.5 $$

However, the diagram drawn at 250 °C shows at pH ~3.5–8 that Fe^II^-monosulfides are hydrolyzed into magnetite upon the chemical equation that I rewrite below:

14 $$ {\mathrm{Fe}}^{\mathrm{II}}{\mathrm{S}}_{\left(\mathrm{s}\right)}+4/3{\mathrm{H}}_2{\mathrm{O}}_{\left(\mathrm{l}\right)}\to 1/3{\mathrm{Fe}}^{\mathrm{II}}{{\mathrm{Fe}}^{\mathrm{II}\mathrm{I}}}_2{\mathrm{O}}_{4\left(\mathrm{s}\right)}+{\mathrm{H}}_2{\mathrm{S}}_{\left(\mathrm{dis}\right)}+1/3{\mathrm{H}}_{2\left(\mathrm{g}\right)}\kern2em {250}^{{}^{\circ}}\mathrm{C},\mathrm{pH}\ 3.5-8 $$

This hydrolysis of ferrous monosulfides can explain the formation of magnetite, H_2_S and H_2_ at pH & T which correspond to the VDVF.

Indeed, the highest temperature Von Damm fluids of the East Summit vent are blown at 226 °C, pH ~5.6, [H_2_]~17 μmol/L, [CH_4_]~2.67 mmol/L (McDermott et al. 2015), [CO_2_]~2.8 mmol/L, [H_2_S] ~3 mmol/L and nmol/L of dissolved C_2_H_6_, C_3_H_8_, n-C_4_H_10_, i-C_4_H_10_ (Table 1, p92 in McDermott 2015). The VDVF field is located on a peridotite-gabbro basement. Gabbroic rocks are known to contain sulfur compounds and therefore H_2_ may originate from the anoxic hydrolysis of ferrous monosulfides as written above. The host rocks of the VDVF, as described in Hodgkinson et al. (2015) & Hodgkinson (2015), contain a serpentinized peridotite with magnetite, Cr-spinel and pyrrhotite (p123 in Hodgkinson 2015). Many associations of talc, with the disulfides chalcopyrite CuFeS_2_, pyrite FeS_2_, and the monosulfides sphalerite (Zn,Fe)S, galena PbS, bornite Cu_5_FeS_4_ are described. *“Sulphides in active chimneys consist of chalcopyrite, pyrite, sphalerite and galena…Sphalerite…occurs…with chalcopyrite or…disseminated in talc along with euhedral cubes of pyrite. Clusters of sulphides at chimneys from the main cone always contain chalcopyrite or pyrite, along with sphalerite or galena, i.e. galena and sphalerite grains are not observed by themselves.”* (p140 in Hodgkinson 2015). Thus, it seems that a relation between iron monosulfides and disulfides, as the one written above, can lead to the high concentration of H_2_S observed in the VDVF and to the attendant H_2_. Therefore, as I write in Bassez (2017a, b): *“Consequently, as I proposed earlier (Bassez* 2013*; Bassez* 2016a, b*), it may be suggested that it is the hydrolysis/oxidation of ferrous sulfide in the absence of oxygen…and not the hydrolysis/oxidation of ferromagnesian silicate, which contributes to explain the low values 3-4 and the H*_*2*_*S concentration between 1.2 and 11 mmol/kg, observed at all high T, low pH fields.”*

FeS hydrolysis is highly endothermic but can proceed within the heat of hydrothermal water complemented by the heat produced by the hydrolysis of Mg-olivine which is exothermic (Bassez 2017a, 2017b). Mg-olivine leads to the serpentine chrysotile which leads to talc. Talc can form in a solution supersaturated in silica by the way of a reaction which is calculated slightly exothermic at 25 °C. Since silica can dissolve at 226 °C & 23 MPa which is the hydrostatic pressure at the location of the 2350 m deep VDVF, water at the VDVF can be supersaturated in silica and talc forms easily in these T&P values which are those of high-subcritical water as discussed above. Therefore, it is plausible that the VDVF sulfides contribute to the great amount of H_2_ & H_2_S, and to the decrease of pH compare to Lost City where most probably only ferromagnesian silicate hydrolysis occurs. This hypothesis of sulfide hydrolysis within the heat of Mg-olivine hydrolysis confirms the observation of mono- and di-sulfides disseminated inside talc. However, if the process of monosulfide hydrolysis at ~226°C is plausible for the VDVF, magnetite deposits should be observed inside the mound below the VDVF as I discussed for the TAG and Rainbow fields in Bassez (2017a, b). A search for magnetic anomalies could be conducted at the VDVF.

A recent experiment reports analyses of micrometer-sized inclusions of the 3.7 Ga old Isua Supracrustal belt, West Greenland (Hassenkam et al. 2017). The high-resolution observations with atomic force microscopy coupled to infrared spectroscopy, AFM-IR, show absorption spectra with a spatial resolution of 10 nm and the analysis concludes in the presence of functional organic groups composed of the elements C, H, O, N and perhaps P.

Oil of different origins seem to appear in fluid inclusions. Oil-bearing fluid inclusions are observed in the ~3 Ga quartz sandstones of Pilbara and Kaapvaal cratons, as well as in the early Paleoproterozoic Huronian Supergroup of the Superior craton, Canada. *“Bituminous nodules...occupy intergranular pore space...and contain remnants of radioactive detrital grains.”* (Dutkiewicz et al. 1998). The observation that radioactivity occurred in these quartz sandstones can be associated to the above described prebiotic synthesis experiment which shows formation of organic compounds with gamma radiation excitation on CO, N_2_ and H_2_O. Indeed, when (CO_2_, CH_4_, N_2_, H_2_O) fluid inclusions are located in appropriate distances of radioactive rocks, gamma emission can most probably excite the gaseous mixture and lead to the synthesis of macromolecules composed of amino acids. The yellow-brown product that is observed with proton irradiation (Kobayashi et al. 1998; Bassez et al. 2012) is most probably the same with gamma radiation, since the *G*-value of glycine is the same for 3 MeV proton and low-dose rate gamma rays. Thus, these inclusions may have been a cradle for the formation of macromolecules of amino acids which were dissolved in the aqueous fluid or aggregated by heat as in an experiment conducted at 300 °C by Kurihara et al. (2012).

#### Conclusion on Geobiotropy inside Fluid Inclusions

Therefore, both heat and gamma ray excitation can lead to the formation of prebiotic molecules inside fluid inclusions, when N_2_ is present. At ~350°C, hydrogenation of CO_2_ produces CO and hydrogenation of CO produces CH_4_. The intermediate CO between CO_2_ and CH_4_ is usually not mentioned. However, it is important to notice that CO can be present and trigger the prebiotic syntheses cited above, as in Sabatier-Senderens/Fischer-Tropsch combined to Haber-Bosch reactions or in excitation by the particles of interaction (as gamma rays) and matter (as helium nuclei) contained in cosmic radiation. Nitrogen is present in crustal rocks and may originate either from the atmosphere or from volcanic processes. Many ammonium-bearing minerals are known (Berg et al. 2016). An analysis of nitrogen in fluid inclusions trapped inside quartz of the 3.0–3.5 Ga Dresser Formation, Warraoona Group in the North Pole Dome area of Pilbara craton, Western Australia, concludes that *“the partial pressure of N*_*2*_ *of the Archean atmosphere was lower than 1.1 bar, possibly as low as 0.5 bar”* (Marty et al. 2013). Modern pN_2_ is ~0.8 bar. Many natural inclusions with various amounts of CO_2_, CH_4_, H_2_, N_2_, H_2_O are observed. CO may form by hydrogenation of CO_2_, and CH_4_ may form through Sabatier-Senderens or Fischer-Tropsch reactions of CO and H_2_, both hydrogenations occurring at approximately the same temperature depending on the catalysts. As shown by Sabatier and Senderens (1902) the amount of CO versus CH_4_ depends very much on the relative abundance of CO_2_ and H_2_ and on the catalysts. Therefore, observations of inclusions with CO_2_, CH_4_, N_2_, H_2_O, mean that H_2_ and CO were present earlier and that prebiotic molecules of biological interest such as macromolecules of amino acids could form. Such inclusions may be located near radioactive rocks or not, inside the chert siderite and hematite of the BIFs. The mineral stilpnomelane, K(Fe^II^,Mg,Fe^III^)_8_(Si,Al)_12_(O,OH)_27_, observed in BIFs, contains the element potassium and consequently the radioactive isotope ^40^K, with a half-life of 1.25 Ga, which desintegrates into the excited ^40^Ar* argon, which returns to its ground state ^40^Ar with emission of a 1.46 MeV gamma radiation. Nitrogen containing organic matter could be searched in the environment of stilpnomelane. And since the formation of organic matter requires carbon from carbon dioxide, the search for prebiotic organic matter requires also locations where carbonates, siderite, or carbon which can be remnant of siderite, are present.

As a conclusion, Fig. 1 gives a summary for the above proposed synthesis of the minerals observed in BIFs and most probably in other hydrothermal silicate rocks, and for the prebiotic chemistry which can occur in symbiosis with the synthesis of ferric compounds, to form macromolecules of amino-acids. I place the emphasis on the consideration of the T&P values of high-subcritical water, which can generate high silica dissolution and oxidation of Fe^II^ into Fe^III^ at high pH, followed by the hydrolysis of fayalite which leads to the release of H_2_. CO_2_ and H_2_ react also at the T&P values of high-subcritical water to trigger the formation of CO and organic molecules. H_2_ is the link between geochemistry and organic chemistry in the process of geobiotropy where the rock evolves towards organic molecules of biological interest in an environment of anoxic high-subcritical water.

## Experimental Preliminary Results

A first set of analyses is undertaken on a sample from the 899 m long BARB3 ICDP drill core in the ~3.4 Ga old Buck Reef Chert of the Barberton Greenstone Belt, South Africa (Arndt et al. 2012, Hofmann et al. 2013). I choosed this Archean rock for the current certainty that no oxygen was present neither in the atmosphere nor in the oceans at the age of formation which is far away from the Great Oxidation Event. I conducted the analyses on March 22^nd^, 2018, in the department of Geology of the University of Johannesburg, with the thin section 23B that Axel Hofmann very kindly provided, and with a Raman Lab Witec Alpha300 instrument composed of a confocal microscope coupled to a Raman spectrograph. The excitation radiation is emitted from a Nd-YAG laser operating at the green 532 nm wavelength. The spectrum of Fig.2 is obtained with a laser power of ~2.5 mW measured at the sample and it is collected with a 600 grooves/mm grating. The acquisition time is 10 times 1 s. The thin section has not been carbon coated.

I assign the spectrum of Fig.2 to α-quartz and siderite as follows. Wavenumbers values are obtained with the graph software of the Witec Project version 6. They are compared mainly to the RRUFF (2018) spectra and raw data which were obtained with a 532 nm laser. The Raman-active modes A and E are assigned mainly with the help of Nasdala et al. (2004), Rousseau et al. (1981), Kingma and Hemley (1994), Blanchard et al. (2009), Boulard et al. (2012), and refs herein.

Sixteen Raman-active vibrations of α-quartz are described in Kingma and Hemley (1994) with their corresponding A and E modes. The RRUFF (n.d.) spectrum of quartz from Bergamo, B, shows 13 of them. They are described in Table 3 with their relative intensities within parentheses. Despite the fact that Raman-active vibrations may have strong directional dependence (Nasdala et al. 2004) several of the Bergamo α-quartz Raman modes are recognized in the Buck Reef Chert, BRC, sample considering both wavenumbers and relative intensities (Fig. 2 and Table 3). The strongest BRC peak at 468 cm^-1^ is assigned to the α-quartz Raman mode A_1_ which corresponds to the symmetric Si-O-Si stretching vibration. The B 510 cm^-1^ is hidden at the base of the BRC 468 cm^-1^ which appears asymmetrical. The 1069 cm^-1^ E mode of α-quartz (not in Bergamo but described in Kingma and Hemley 1994) and the B 1083 cm^-1^ are overlapped by the strong siderite 1090 cm^-1^ mode A_1g_.

**Table 3:** Assignment of the Raman spectrum of the Buck Reef Chert sample 23B, of Fig.2

Raman wavenumbers cm^-1^			Raman modes
Buck Reef Chert *	Bergamo α-quartz *	Connecticut siderite *	
130 (3)	129 (19)		E
188 (5)		185 (4)	E_g_
207 (5)	207 (18)		A_1_
	267 (3)		E
286 (5)		286 (5)	E_g_
359 (1)	357 (6)		A_1_
	395 (1.5)		E
405 (0.2)	404 (1)		E
468 (20)	466 (73)		A_1_
	510 (3)		E
	697 (0.6)		E
736 (1)		729 (1)	Eg (ν_4_)
809 (0.5)	810 (3)		E
	1083 (2)		A_1_
1090 (37)		1086 (29)	A_1g_ (ν_1_)
1165 (0.1)	1161 (1)		E
	1233 (0.2)		E
1730 (1)		1728 (1)	ν_1_ + ν_4_

*The values within parentheses indicate the relative intensities of the peaks, that I calculated in their own spectrum. In order to compare the intensities of Buck Reef Chert with Bergamo quartz, it is necessary to multiply the BRC values by 5

The identification of the other lines of the Buck Reef Chert sample, as shown in Table 3, are based mainly on the Raman RRUFF (n.d.) spectrum of a Connecticut, C, siderite which is the siderite RRUFF (n.d.) sample with the highest content in Fe. Wavenumbers and relative intensities match very well. Wavenumbers correspond also to those of the Raman-active modes A_1g_ (symmetric single vibration or single degenerate) and E_g_ (doubly degenerate) of siderite, described in Blanchard et al. (2009). The peak of very low intensity at 736 cm^-1^ corresponds to the Raman mode Eg of the bending vibration in the plane of the -CO_3_ group. It appears slightly asymmetrical as expected for Fe-bearing carbonates (Boulard et al. 2012). The strong peak at 1090 cm^-1^ is due to the symmetric in-plane stretching vibration of the -CO_3_ group which appears as the Raman mode A_1_g. The mode Eg of the asymmetric stretching vibration, which appears between 1442 and 1446 cm^-1^ in the Raman spectra of carbonates, as for smithsonite ZnCO_3_ and spherocobaltite CoCO_3_, is not detected in BRC. Since this mode of vibration is of very low intensity in the case of Fe-rich carbonates (Boulard et al. 2012), the absence of this peak confirms the detection of siderite. Finally, the low intensity peak at 1730 cm^-1^ corresponds to a combination of the A_1g_ symmetric stretching with the E_g_ bending mode in the plane of the CO_3_ group.

The Buck Reef Chert 405 cm^-1^ could indicate the presence of hematite (Hanesch 2009; Oh et al. 1998). However, neither the strong peak of hematite at ~1320 cm^-1^ nor the medium peaks at 225 cm^-1^ and ~295 cm^-1^ are present. Another weak and broad band appears centered at 2902 cm^-1^. It is the region of C-H stretching vibrations. More investigations are needed. Therefore, the analyzed location of the Buck Reef Chert sample shows the presence of crystalline α-quartz and siderite. Amorphous silica is absent. Another location of the colorful thin section will most probably show signs of hematite, which in association with quartz and siderite, can be at the origin of prebiotic molecules as described above.

## Conclusion

With this article, I try to set the emphasis on the state of water which is basic (pH 9.5–14) and high-subcritical (300 °C–350 °C, 10–25 MPa). I show that these conditions transform ferrous iron into ferric iron in the absence of oxygen and that Fe^III^-oxides and Fe^III^-silicates are synthesized. This anoxic synthesis of Fe^III^-compounds is applied to the minerals of Banded Iron Formations in an attempt to explain their origin. The interaction between basic silica-undersaturated hsc water and fayalite/ferrosilite at depth inside the Earth crust can lead to the minerals of BIFs. Hematite is a primary product while magnetite is secondary. Fe^III^-greenalite is a primary product as minnesotaite and possibly riebeckite and stilpnomelane. The formation of amorphous silica versus crystalline quartz is dependent upon this high-subcritical state of water and its pH. The type of minerals which forms depends on the content in silica. In high silica content, the Fe^III^-greenalite dehydrated products such as minnesotaite, stilpnomelane, riebeckite may dominate and incorporate ferric iron.

In the above chapter on the formation of BIFs, I concluded in the necessity of water showing four characteristics at a specific kairos in space and time: high-subcritical state, high pH, undersaturated in silica and interacting with iron-olivine and -pyroxenes. As a global conclusion for the formation of BIFs and molecules of life inside fluid inclusions, I introduce now the necessity of four chemical processes which all occur in high-subcritical water. First, at high-subcritical conditions of water, and not above, ferrous iron transforms into ferric iron with release of H_2_ at high pH. This is observed from E-pH diagrams. Second, also at high-subcritical conditions of water, and not above, silica dissolves in water. This is observed from a solubility diagram showing a transition at the critical point of water. The third process is the interaction of silica-undersaturated hscw with Fe^II^-silicates to produce the ferric compounds of BIFs and H_2_, and the fourth process is the hydrogenation of CO_2_ in hscw to produce CO, an essential molecule for prebiotic synthesis. In their interaction with silica-undersaturated hscw, Fe^II^-silicates produce silica. Near supercritical water can thus become supersaturated in silica and dehydration of the hydrolysis product, greenalite, can proceed into minnesotaite. The hydrolyses of fayalite and ferrosolite in carbonated near-supercritical water lead to the main products, ferric hydroxide, hematite, silica, siderite, greenalite and minnesotaite, which are the minerals of the banded iron formations. Further dehydration can most probably lead to riebeckite and stilpnomelane. And magnetite can form in the transformation of siderite. Silica which is formed at these T&P near 374 °C and 22 MPa and then quenched in cool ocean water can be structured as a glass. As a consequence, water near its supercritical point shows properties which trigger two effects complementing each other: the formation of ferric compounds and of dissolved silica leading to dehydrated ferric silicates. Following the emission of H_2_ in the hscw-rock interaction, the formed CO can react at the same temperature with H_2_ and N_2_/NH_3_ to form molecules of biological interest such as aggregates of amino-acids which are synthesized in the above described laboratory experiments.

Therefore, anoxic alkaline water in its high-subcritical state appears, to my knowledge, as an essential component for the chemical synthesis of minerals in Banded Iron Formations and for the connected geobiotropic chemistry inside fluid inclusions.
